# Utility of plasma NGAL for the diagnosis of AKI following cardiac surgery requiring cardiopulmonary bypass: a systematic review and meta-analysis

**DOI:** 10.1038/s41598-022-10477-5

**Published:** 2022-04-19

**Authors:** Hayley Sharrod-Cole, Jonathan Fenn, Rousseau Gama, Clare Ford, Ramesh Giri, Heyman Luckraz

**Affiliations:** 1 Clinical Biochemistry, Black Country Pathology Services, Wolverhampton, West Midlands UK; 2 School of Medicine and Clinical Practice, Wolverhampton University, Wolverhampton, West Midlands UK; 3 Cardiac Anaesthesia, Royal Wolverhampton NHS Trust, Wolverhampton, West Midlands UK; 4 Cardiac Surgery, Royal Wolverhampton NHS Trust, Wolverhampton, West Midlands UK; 5 Department of Clinical Biochemistry, New Cross Hospital, Wolverhampton, WV10 0QP UK

**Keywords:** Biochemistry, Cardiology, Health care, Nephrology

## Abstract

The objective of this study was to assess the diagnostic value of plasma neutrophil gelatinase-associated lipocalin (pNGAL) for the early diagnosis of acute kidney injury (AKI) in adult patients following cardiac surgery requiring cardiopulmonary bypass (CPB). Electronic databases and other resources were systematically searched for relevant studies. Risk of bias was assessed using the Quality Assessment for Diagnostic Accuracy Studies 2 (QUADAS-2) tool. Studies were assigned to a sub-group based on the timing of the pNGAL sample in relation to the cessation of CPB. These were < 4 h, 4–8 h, 12 h or 24 h post-cessation of CPB. Summary values for sensitivity and specificity were estimated using the hierarchical summary receiver operator characteristic (ROC) curve model. A random-effects meta-analysis of each pair of sensitivity and specificity estimates from each included study was performed. In total, 3131 patients from 16 studies were included. When taken at 4–8 h following CPB, pNGAL had superior performance for the diagnosis of AKI in the defined population when compared to earlier and later time points. Prediction regions and confidence intervals, however, demonstrated significant variability in pooled estimates of sensitivity and specificity. This is likely due to population and study design heterogeneity, lack of standardisation of assays and thresholds, and inability to distinguish the different molecular forms of NGAL. In conclusion, the diagnostic utility of pNGAL in this clinical setting is inconclusive and large individual studies of representative populations of cardiac surgery patients using assays that specifically detect NGAL in its monomeric form are required.

## Introduction

Acute kidney injury (AKI) is a common complication of cardiac surgery^1^ and is associated with significant mortality, morbidity and increased health care costs^2^. Studies have reported that AKI, and more specifically AKI requiring renal replacement therapy (RRT) is associated with further increased mortality and longer intensive care unit (ICU) and total hospital length of stay (LOS)^3–7^. Patients undergoing cardiac surgery requiring cardiopulmonary bypass (CPB) are at even greater risk of developing AKI. The mechanism of kidney injury following CPB is multifactorial. Contributing factors include ischemia–reperfusion injury, reduced cardiac output, renal vasoconstriction, CPB hypothermia and rewarming, CPB-induced systemic inflammatory response leading to interstitial inflammation and coagulopathy, which in turn can lead to CPB-related embolization^8–12^. The reported incidence of post-cardiac surgery AKI varies depending on the definition of AKI and the clinical profile of patients studied. A large meta-analysis reported a rate of CPB associated AKI of 18.2% which is associated with a greater than twofold increase in mortality^13^.

In clinical practice, the diagnosis of AKI largely relies on the detection of an acute rise in serum creatinine and/or a reduction in urine output, both of which are considered suboptimal markers of AKI. Serum creatinine is affected by many factors including age, sex, race, body surface area, diet, diabetes, liver disease, drugs and laboratory analytical methods which compromise its performance as a diagnostic test for AKI^14^. Serum creatinine is a relatively late marker of AKI and up to 50% of glomerular function is lost before the serum creatinine is elevated above the reference range^15^. Neutrophil gelatinase-associated lipocalin (NGAL) is expressed and secreted by kidney cells soon after renal insult, including post-operative ischemia following CPB. NGAL concentrations rise rapidly, within 2–6 h, and are detectable in plasma when AKI is potentially limitable or reversible^16,17^ A meta-analysis of 307 studies involving 1200 patients, reported an area under the receiver operator characteristic curve (AUROC) of plasma NGAL (pNGAL) for predicting AKI post-cardiac surgery as 0.78 (95% confidence interval 0.67 to 0.87). Plasma NGAL is therefore considered to be a sensitive and specific early marker of AKI and can be detected up to 48 h before a diagnostic rise in serum creatinine^17^. A meta-analysis of 53 studies with over 7000 patients undergoing cardiac surgery reported that pNGAL was predictive of AKI and its severity, with an AUROC of 0.82–0.83^18^. This meta-analysis did not specifically review a patient cohort undergoing CPB.

In 2018, the AKI-diagnostics project reviewed the diagnostic performance of several potential tests including pNGAL for AKI in an ICU setting. Whilst a subgroup of post cardiac surgery patients was considered, the number of studies included was small and was not specifically defined as cardiac surgery requiring CPB. The review concluded that whilst pNGAL has the potential to add value, results of the analysis were highly uncertain, largely due to heterogeneity between studies^19^. A health technology assessment of pNGAL (and other biomarkers) in AKI in critically ill patients in 2019 similarly advised cautious interpretation of results due to heterogeneity between studies but concluded that future studies should evaluate the targeted use of biomarkers such as pNGAL within specific patient populations^20^. We therefore sought to quantitatively summarize published studies to evaluate the diagnostic accuracy of pNGAL for AKI in a specific clinical setting of adult patients who have undergone cardiac surgery requiring CPB.

## Methods

The systematic review was registered with the International Database of Prospectively Registered Systematic Reviews (PROSPERO; registration number CRD42021261676) and conducted in accordance with the PRISMA guideline for reporting of systematic reviews (Supplementary Information)^21^.

### Identification of studies

A comprehensive electronic search was carried out through PubMed, Cochrane database, Scopus, and ClinicalTrials.gov (http://clinicaltrials.gov/) up until June 2021. The search terms used in PubMed were: (heart OR cardiac OR aort* OR valv* OR thoracic) AND surg*) OR ‘valve replacement*’ OR ‘bypass*’ OR ‘CABG’ OR ‘extracorporeal circulation’ OR ‘on pump’ OR ‘Cardiac Surgical Procedures’) AND (‘neutrophil gelatinase-associated lipocalin’ OR NGAL OR ‘LCN2 protein, human’) AND (‘diagnostic accuracy’ OR ‘sensitivity’ OR ‘specificity’ OR ‘PPV’ OR ‘NPV’ OR ‘positive predictive value’ OR ‘negative predictive value’) . In the Cochrane library, Scopus and ClinicalTrials.gov, a similar strategy was used. In addition, abstracts from meetings and reference lists of eligible papers or related reviews were searched manually to identify additional relevant studies.

### Inclusion and exclusion criteria

The inclusion criteria for studies were: (i) adult cardiac surgery cohort requiring CPB; (ii) measurement of pNGAL for the early diagnosis of AKI (within 24 h) after cardiac surgery; (iii) provision of data from which true-positive (TP), false-positive (FP), false-negative (FN) and true-negative (TN) could be identified or calculated; (iv) AKI clearly defined by acceptable methods- preferably by KDIGO, RIFLE or AKIN criteria^22–24^ and (v) those published in English. Exclusion criteria were: (i) studies with duplicate data reported in other studies; (ii) sample size less than 25; (iii) timing of pNGAL measurement not clearly defined; (iv) inclusion of paediatric patients within the cohort; (v) more than 20% ‘off-pump’ patients included in the cohort; (vi) insufficient diagnostic accuracy data available.

### Study selection and data extraction

One reviewer (HSC) screened the titles and abstracts of all citations to judge eligibility based on the inclusion and exclusion criteria. For citations that could not be evaluated through the titles and abstracts, full texts were retrieved for thorough evaluation.

A second reviewer (JF) second checked all prospective citations for eligibility. Full-text copies of all potentially relevant reports were retrieved and assessed for inclusion by both reviewers (HSC and JF).

One reviewer (HSC) extracted the data from each study. This was checked for accuracy by the second reviewer (JF). Any discordance was then checked by the first reviewer. The following information was recorded from each selected study (i) basic characteristics of studies: name of the first author, year of publication, sample size, country; (ii) characteristics of cohort: AKI diagnosis criteria, number of ‘off-pump’ patients, number of patients who developed AKI; (iii) measurement of pNGAL: specimen type, analytical method, pNGAL test cut-off and the timing of sample collection; (iv) the criteria for the diagnosis of AKI; (v) study outcomes: test sensitivity and specificity and or true positive (TP), false positive (FP), false negative (FN), true negative (TN), positive predictive value (PPV), negative predictive value (NPV) and AUROC.

### Assessment of the risk of bias

The Quality Assessment of Diagnostic Accuracy Studies version 2 (QUADAS-2) tool was used to assess the risk of bias^25^. The following items were evaluated: patient selection, interpretation of the index test, appropriateness and interpretation of the reference standard, flow of patients and timing of tests. The applicability of each study to the question under review was also assessed to consider whether the procedures employed in a study would differ significantly from those employed in real clinical practice. Each item was scored as either low risk of bias, unclear risk of bias or high risk of bias. Two reviewers (HSC and JF) assessed the risk of bias.

Studies identified to pose a high risk of bias were not excluded from the meta-analysis, but the findings were instead interpreted in light of the bias, which is in keeping with good practice.

### Data analysis

For each study, sensitivity, specificity, prevalence, PPV, NPV, TP, FP, FN and TN cases were recorded. If a study lacked the mandatory diagnostic accuracy data, the TP/FP/FN/TN according to the following formulae: sensitivity = TP/ (TP + FN), specificity = TN/(FP + TN), AKI + non-AKI = TP + FP + TN + FN were calculated and entered into a 2 × 2 table.

The diagnostic data were entered into Review Manager software (RevMan version 5.4, Nordic Cochrane Centre, Copenhagen) to generate forest plots of sensitivity and specificity. The odds ratio was used for the synthesis and presentation of results.

To estimate the summary values for sensitivity and specificity, and their 95% confidence and prediction regions, a random-effects meta-analysis odds ratio (OR) was performed using the hierarchical summary ROC (hSROC) model implemented in STATA^®^ software version 16.1 (StataCorp LP, College Station, TX, USA) using the METANDI command. This model is described in the Cochrane Handbook for Systematic Reviews of Diagnostic Test Accuracy for comparisons of test accuracy when there is variability in threshold between studies and is preferred as it takes into account both sensitivity and specificity measures and the correlation between them, assumes that thresholds vary between studies and incorporates variability within and between studies^26^. In the presence of heterogeneity, which was expected in this review, a random-effects meta-analysis weights the studies relatively more equally than a fixed-effect analysis.

In accordance with the STATA requirements, meta-analyses were performed only when data from four or more studies were available. For studies that reported multiple time points, each was assigned to a group based on the timing of the pNGAL sample in relation to the cessation of CPB. These were < 4 h, 4–8 h 12 h or 24 h post-cessation of CPB. A separate meta-analysis was performed for each time point. Subgroup meta-analysis was also performed based on whether a point of care test (POCT) or laboratory-based method was used.

Heterogeneity was assessed by visual inspection of the forest plots and of the size of the prediction region in the hSROC plots. The index of variability (*I*^2^*)* statistic and chi-squared test statistic were used to approximate proportion of total variability in point estimates that could be attributed to heterogeneity, although the limitations of this approach are discussed. An *I*^2^ > 50% with a p-value < 0.05 from the chi-squared test was indicative of moderate heterogeneity^27^.

### Sensitivity analysis

Studies identified to have a high risk of bias were individually removed from the meta-analysis and data to determine the effect on the summary points and heterogeneity.

## Results

### Summary of included studies

**Figure 1:**
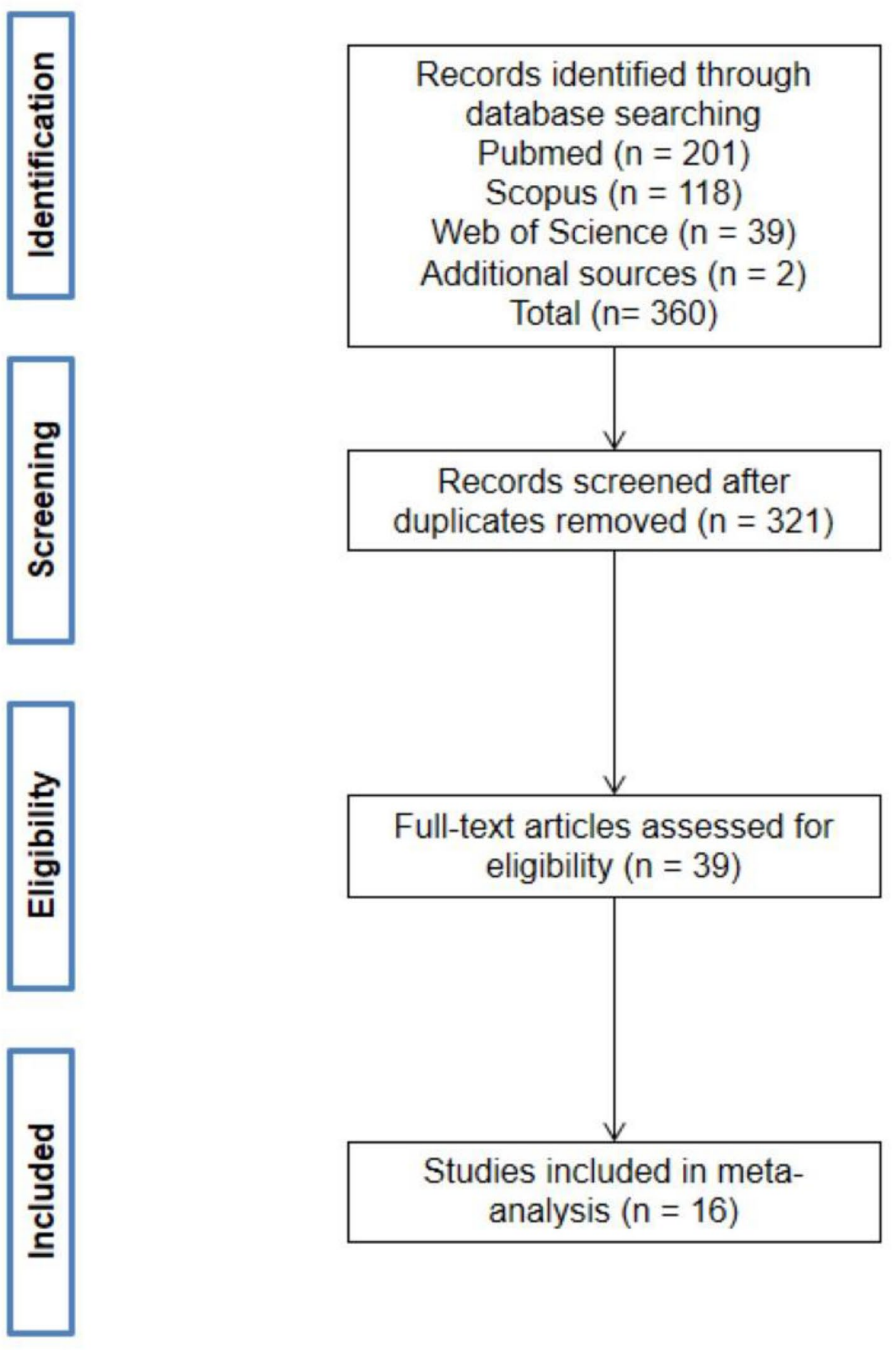
Flow of studies through the selection process.

The literature search yielded 360 records. There were 321 records remaining following removal of duplicates. Following removal of studies that did not meet the inclusion criteria, 39 full text articles were reviewed, and 16 individual studies were included in the final meta-analysis (Fig. 1). The characteristics of the selected studies are presented in Table 1.

**Table 1:** Included studies.

First author, year	Assigned time since CPB cessation (h)	Sample size	AKI Prevalence	TP	FP	FN	TN	pNGAL cut-off (ng/mL)	Sensitivity	Specificity	PPV	NPV	AUROC (95% CI)
Merkle, 2019^42^	< 4	58	0.36	15	3	6	34	226.98	0.71	0.92	0.83	0.85	0.846 (0.727–0.927)
	24	58	0.36	18	8	3	29	115.87	0.86	0.78	0.69	0.91	0.891 (0.781–0.957)
Introcaso, 2018^43^	4–8	69	0.35	18	18	6	27	154	0.76	0.59	0.5	0.81	0.71 (0.60–0.82)
Guerci, 2018^44^	4–8	50	0.36	15	5	3	27	112	0.83	0.84	0.75	0.9	0.9 (0.81–0.98)
Schley, 2015^45^	< 4	110	0.34	28	18	9	55	173.43	0.76	0.75	0.609	0.859	0.81 (0.73–0.90)
	4–8	110	0.34	29	18	8	55	178.20	0.78	0.75	0.617	0.873	0.83 (0.75–0.91)
	24	110	0.34	32	18	5	55	137.61	0.86	0.75	0.640	0.916	0.84
Park, 2015^46^	4–8	112	0.12	8	11	5	88	168.5	0.62	0.89	0.421	0.946	0.81 (0.68–0.95)
Kidher, 2014^47^	< 4	53	0.30	13	4	3	33	150	0.80	0.89	0.765	0.917	0.83 (0.70–0.95)
Ghonemy, 2014^30^	4–8	50	0.34	17	3	0	30	62	0.98	0.92	0.842	0.967	Not stated
	< 4	50	0.34	16	2	1	31	62	0.94	0.94	0.888	0.968	Not stated
Parikh, 2011^29^	< 4	1219	0.05	30	209	30	950	293	0.50	0.82	0.126	0.969	0.7 (SE 0.04)
Perry, 2010^48^	< 4	879	0.09	29	149	46	655	353.5	0.39	0.82	0.163	0.934	0.641 (0.58–0.71)
	24	879	0.09	42	242	33	562	353.5	0.55	0.70	0.148	0.945	0.670 (0.60–0.74)
Haase, 2009^49^	4–8	100	0.46	34	14	12	40	150.0	0.73	0.74	0.708	0.769	0.77 (0.63–0.91)
Haase-Fielitz, 2009^18^	4–8	100	0.23	18	17	5	60	150.0	0.79	0.78	0.52	0.93	0.80 (0.63–0.96)
	24	100	0.23	21	18	2	59	150.0	0.91	0.76	0.53	0.97	0.87 (0.78–0.96)
Perrotti, 2015^50^	24	166	0.31	31	27	21	87	258	0.60	0.76	0.534	0.806	0.7
	12	166	0.31	43	48	9	66	173	0.82	0.58	0.473	0.88	0.7
	4–8	166	0.31	41	48	11	66	155	0.79	0.58	0.461	0.857	0.7
	< 4	166	0.31	28	33	24	81	178	0.54	0.71	0.459	0.771	0.6
Gaipov, 2015^51^	12	60	0.67	21	5	19	15	199	0.52	0.73	0.808	0.441	0.649 (0.507–0.774)
	24	60	0.67	32	11	8	9	175.4	0.79	0.46	0.744	0.529	0.646 (0.504–0.772)
Prabhu, 2010^52^	4–8	30	0.27	8	2	0	20	229	1.00	0.91	0.794	1	0.98
Tuladhar, 2009^53^	< 4	50	0.18	7	14	2	27	426	0.80	0.67	0.333	0.931	0.85 (0.73–0.97)
Othman, 2021^28^	4–8	25	0.28	7	2	0	16	145	1.00	0.89	0.778	1	0.965

A total of 3131 individual patients were included. The studies were split into groups based on the timing of the pNGAL sample; these were < 4 h, 4–8 h 12 h or 24 h post-cessation of CPB. Where this was not clearly stated in the study, a pragmatic approach was taken and agreed by three reviewers (HSC, JF and HL). In the subsequent QUADAS-2 assessment the applicability of the index test to review the question was then judged to be unclear. A total of 8 studies were classified as < 4 h and included 2585 patients, 10 studies were classified as 4–8 h and included 812 patients, two studies classified as 12 h, and six at 24 h comprising 1373 patients (Table 2).

**Table 2:** Summary of included studies.

Timing of pNGAL sample (hrs since CPB)	Number of studies	Number of patients
< 4	8	2585
4–8	10	812
12	2	226
24	6	1373

### Study quality

**Figure 2:**
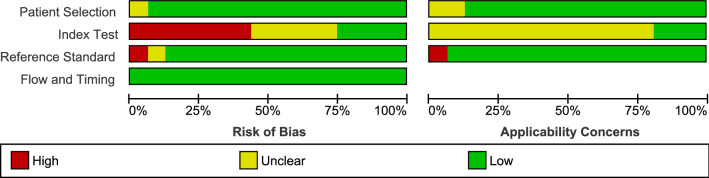
Risk of bias assessment using the QUADAS-2 tool: overall summary.

**Figure 3:**
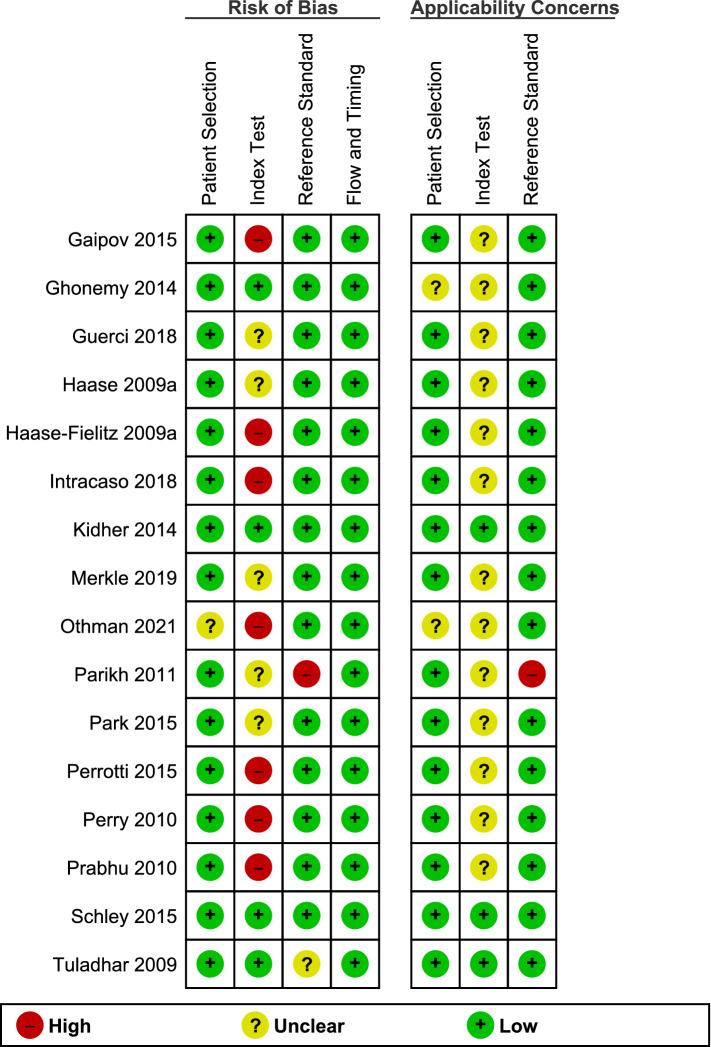
Risk of bias assessment using the QUADAS-2 tool: individual study summary.

Risk of bias was assessed using the QUADAS-2 tool and summarised in Figs. 2 and 3. Most studies (94%) were deemed to be at low risk of bias due to patient selection. One study cohort included only patients who had undergone coronary artery bypass graft (CABG) which may not be representative of all cardiac surgery patients^28^. The main potential source of bias related to blinding. Most studies were assessed as being either at high risk (44%) or unclear risk (31%) of bias for the conduct or interpretation of the index test largely due to the interpretation of the index test (pNGAL) occurring with prior knowledge of the reference standard results. One study was deemed to be at high risk of bias due to the classification of AKI as a doubling of serum creatinine which would exclude early AKI and explains the lower study prevalence of AKI (4.9%) compared to other studies^29^. There were no concerns regarding risk of bias introduced by the flow and timing of samples.

The applicability of patient selection was deemed to be unclear in two studies. In one study, it was not explicitly stated that patients were on CPB, but this was implied in the discussion and conclusions of the paper^30^. The applicability of the index test to review the question was judged to be unclear in most studies (81%) due to the variation in pNGAL thresholds or lack of clarity of timing of sample collections.

### Diagnostic accuracy of pNGAL for identifying AKI in the defined cohort

**Figure 4:**
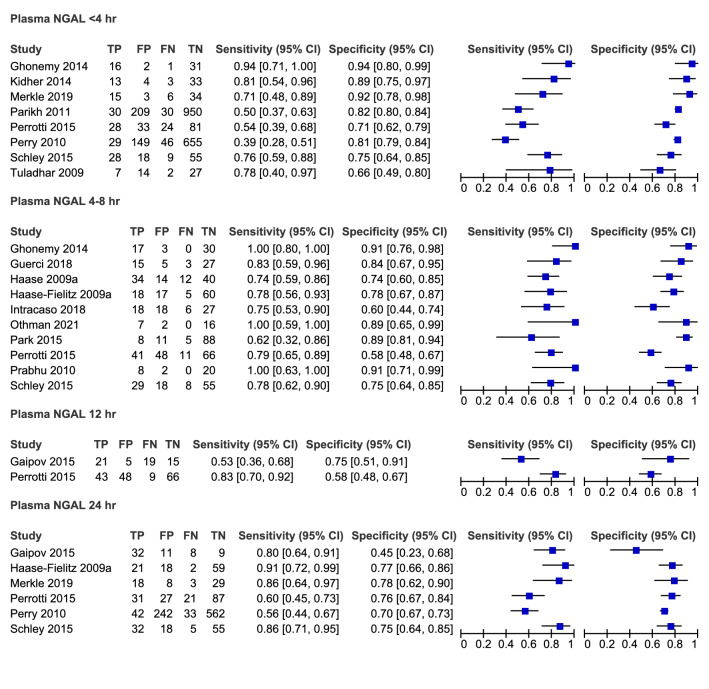
Forest plots of all studies categorised by time post CPB cessation.

The 12 h group was excluded from further analysis due to insufficient study numbers. Individual study sensitivities ranged from 0.39–0.94, 0.62–1.00 to 0.56–0.91 in the < 4 h, 4–8 h, and 24 h groups respectively. Forest plots of sensitivity and specificity for all studies are presented in Fig. 4. The summary estimates of sensitivity were 0.68 (95% CI 0.52–0.8), 0.81 (95% CI 0.74–0.87), 0.78 (95% CI 0.64–0.87) and specificity 0.82 (95% CI 0.75–0.88), 0.8 (95% CI 0.71–0.87), 0.73 (95% CI 0.69–0.77) in the < 4 h, 4–8 h, and 24 h groups respectively (Table 3).

**Table 3:** Summary points and sensitivity analysis.

Time since CPB (h)	Description of studies	No studies	Sensitivity	95% CI	Specificity	95% CI
< 4	All studies	8	0.676	0.521–0.800	0.822	0.753–0.875
4–8	All studies	10	0.814	0.743–0.869	0.801	0.713–0.868
4–8	Othman, 2021 study removed	9	0.802	0.734–0.856	0.791	0.698–0.860
4–8	Haase, 2009 study removed	9	0.831	0.752–0.888	0.810	0.712–0.880
24	All studies	6	0.776	0.638–0.871	0.729	0.685–0.769
24	Perry, 2010 study removed	5	0.810	0.686–0.893	0.744	0.692–0.790
4–8	NGAL ELISA methods only	4	0.986	0.362–0.999	0.869	0.753–0.935
4–8	Triage NGAL methods only	6	0.755	0.680–0.818	0.751	0.642–0.836

**Figure 5:**
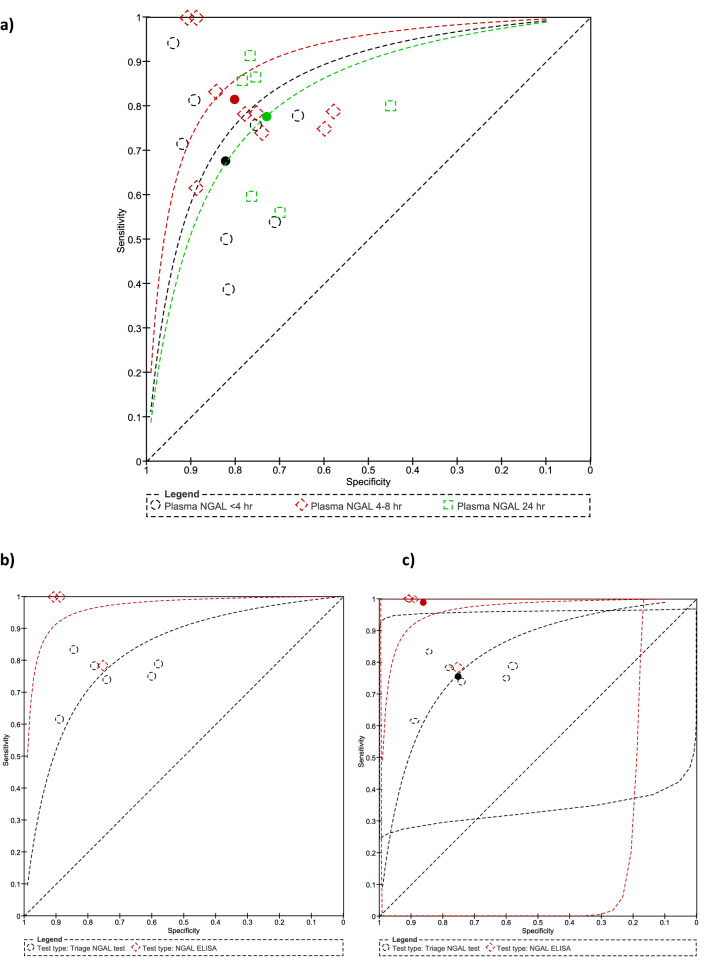
(**a**) Summary ROC plots with hSROC summary points overlaid, (**b**) summary ROC when method type was added as a covariate, (**c**) summary ROC when method type was added as a covariate with hSROC summary points and prediction regions overlaid.

Summary ROC curves (Fig. 5) suggest pNGAL when taken at 4–8 h post-cessation of CPB to be the optimal test. The 4–8 h group was, therefore, subjected to in depth analysis. When the method type (Triage NGAL versus NGAL ELISA) was added as a covariate the shape of the summary ROC further improved. However, when the hSROC analysis was applied, the prediction regions indicated a large degree of heterogeneity in both sensitivity and specificity estimates (Table 3). It is worth noting the individual sample sizes in the four studies in this group were small.

**Figure 6:**
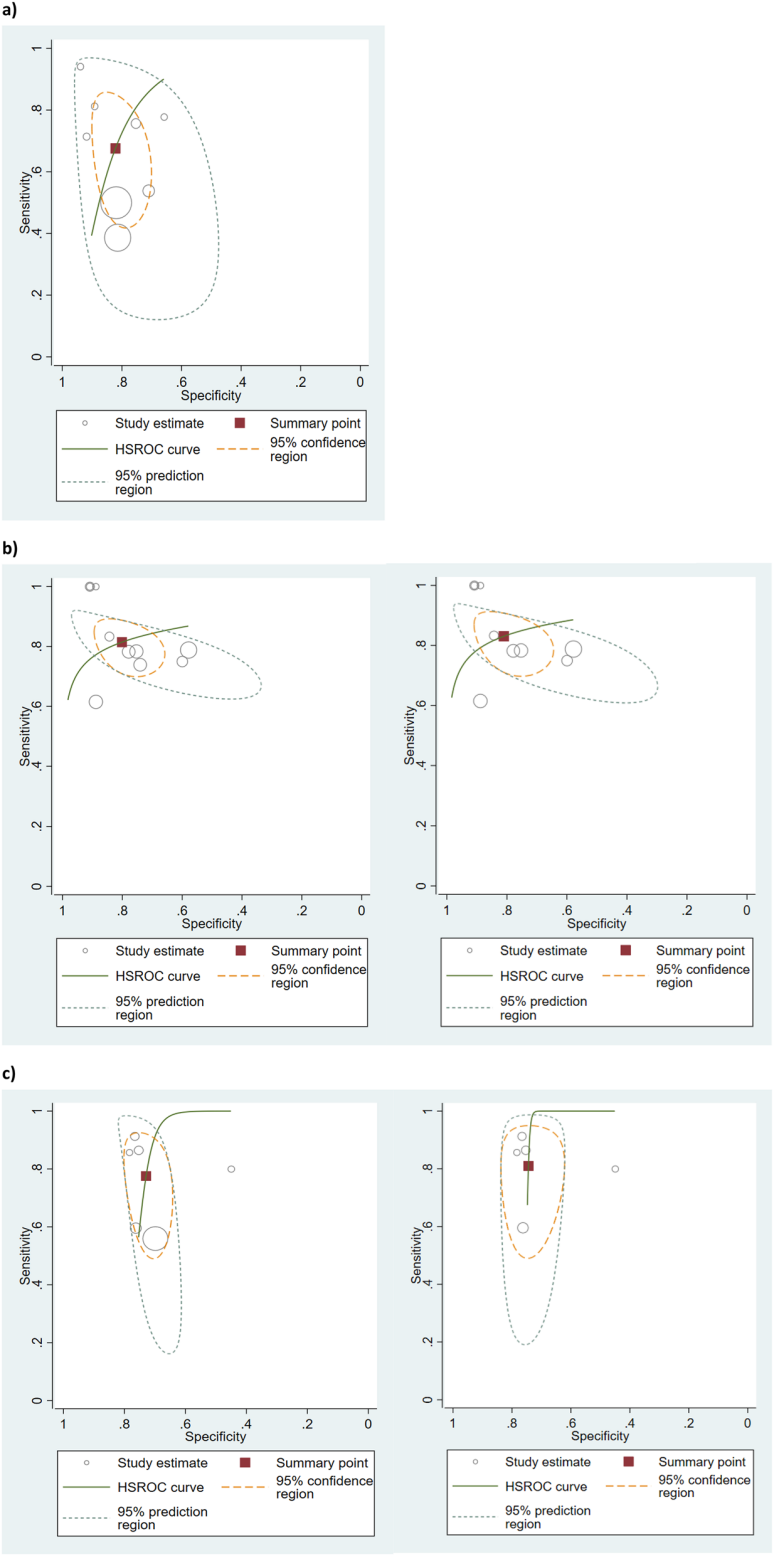
(**a**) hSROC of pNGAL taken at < 4 h post CPB, (**b**) hSROC of pNGAL taken at 4–8 h post CPB: left hSROC including all studies and right hSROC following removal of Haase, 2009 study due to possible incorrect subgrouping, (**c**) hSROC of pNGAL taken at 24 h post CPB: left hSROC including all studies and right hSROC following removal of Perry, 2010 study due to high risk of bias.

Figure 6a–c show the hSROC for each time point with 95% confidence region for the summary operating point and 95% prediction region. At < 4 h, the confidence and prediction regions indicate a greater degree of heterogeneity in sensitivity estimates than in specificity estimates between studies. Specificity estimates were reasonably homogeneous. At 4–8 h, the confidence and prediction regions indicate a greater degree of heterogeneity in specificity estimates than in sensitivity estimates between studies. At 24 h, specificity estimates were reasonably homogeneous but there was a considerable degree of heterogeneity in the sensitivity estimates.

### Heterogeneity and covariates

**Figure 7:**
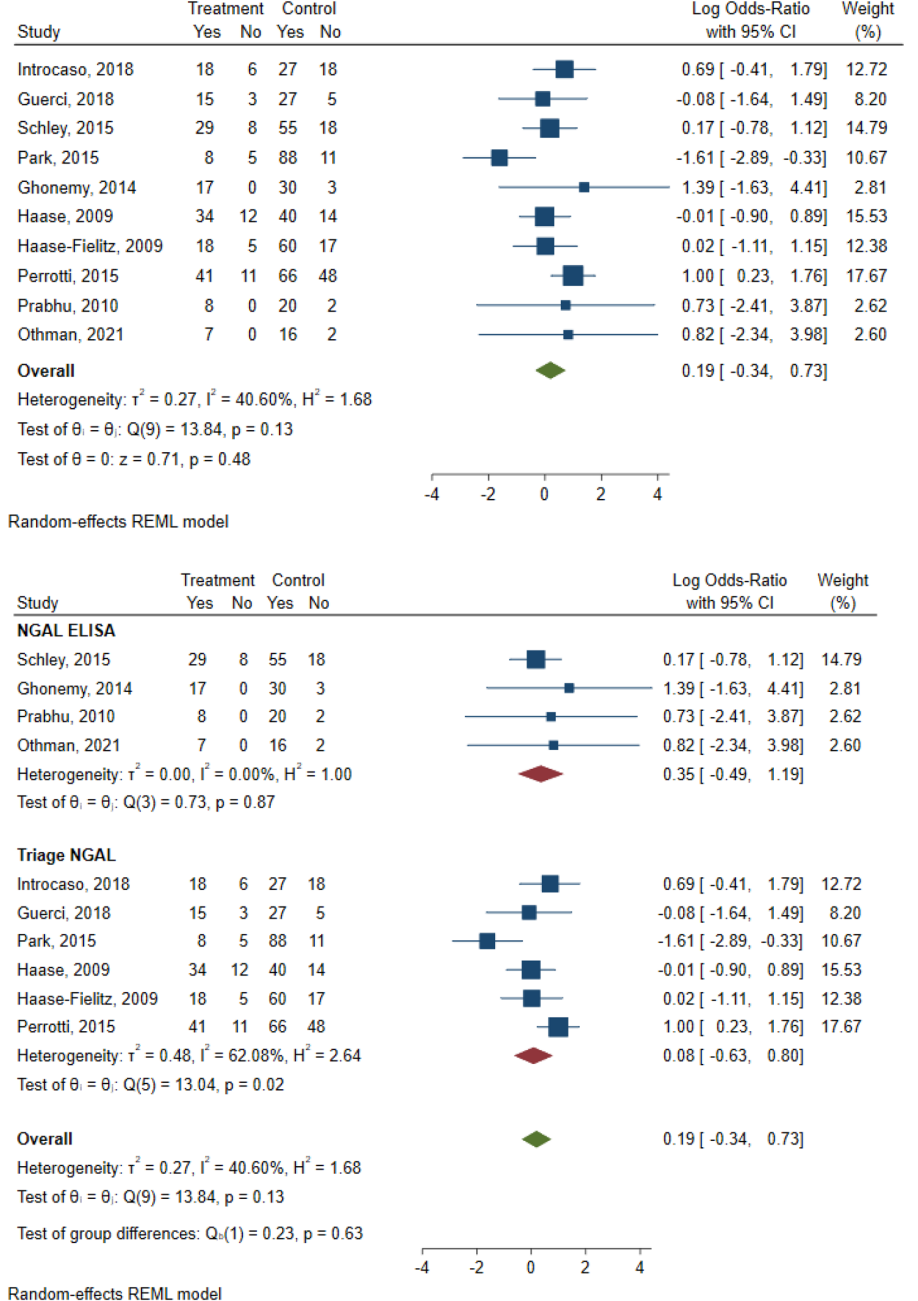
Random effects-meta-analysis of odds ratios and subgroup analysis in the 4–8 h group.

The between-study variation of the effect sizes is evident from visual inspection of the forest plot. In the 4–8 h group the *I*^2^ statistic was 40.6%, indicating mild heterogeneity^27^. Subgroup analysis based on method type showed that there was no observed heterogeneity in the NGAL ELISA group (*I*^2^ = 0%) and moderate heterogeneity (*I*^2^ = 62.1%) in the Triage NGAL test group. The test of homogeneity of study-specific effect sizes was also rejected in the Triage NGAL test subgroup, with a chi-squared test statistic of 13.02 and a p-value of 0.02 (Fig. 7).

### Publication bias

**Figure 8:**
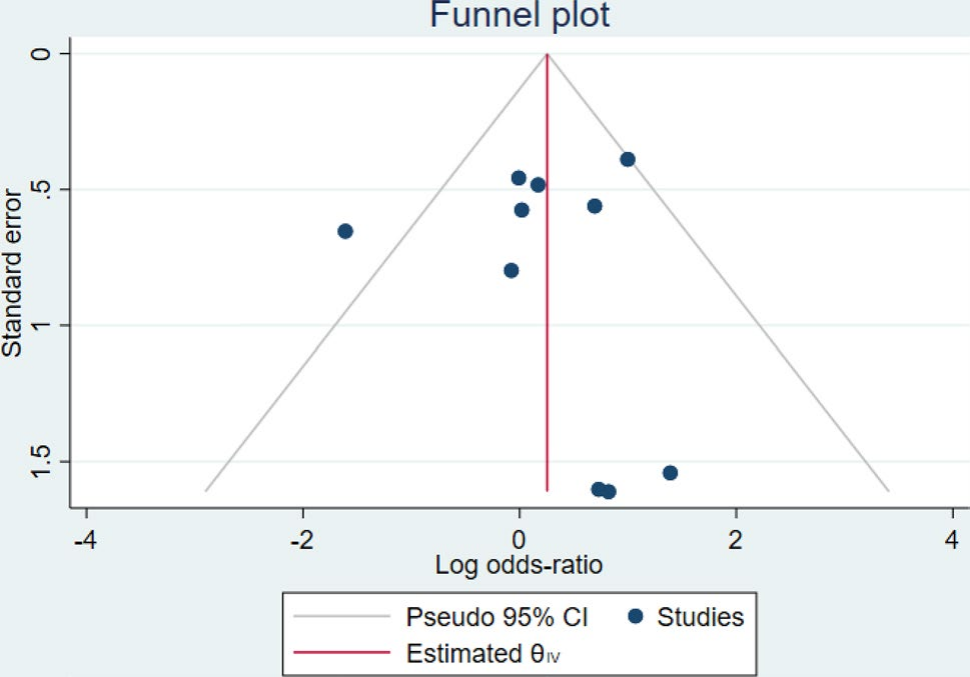
Funnel plot of effect size versus standard error in the 4–8 h group.

Funnel plot asymmetry was evident in the 4–8 h group. There was a clear absence of studies in the lower left portion of the plot (Fig. 8). Although it should be noted that the number of studies is small and therefore funnel plot asymmetry does not necessarily indicate publication bias.

## Discussion

Our meta-analysis of sensitivity and specificity estimates indicates that pNGAL taken 4–8 h following cessation of CPB in cardiac surgery patients is superior to pNGAL taken at < 4 h or 24 h for the early diagnosis of AKI. This is supported by data from the TRIBE-AKI study which reported that pNGAL concentrations peak at 6 h post cardiac surgery^29^. It was expected that the pooled estimates for sensitivity and specificity would be greater at 24 h, however the number of studies in this group was small. In addition, one study in the 24 h group was identified as high risk of bias due to the classification of AKI as a doubling of serum creatinine which would exclude early AKI defined by other standard criteria. The bias is supported by the lower prevalence of AKI (4.9%) compared to reported prevalence of AKI post-cardiac surgery (18.2%)^13^. Exclusion of this study from the meta-analysis improved summary points for sensitivity and specificity but the confidence and prediction regions increased.

The prediction regions and confidence limits at all time points were large due to considerable clinical and statistical heterogeneity observed across studies and the limited number of studies available for subgroup analyses. Surgery type, and patient related risk factors including age, sex, diabetes mellitus, basal renal function and congestive heart failure, contribute to the complex relationship between co-morbid pathophysiology and CPB as major causes of AKI in the population studied^5,24,31^. There was, however, significant variation between individual studies in patient inclusion criteria and risk factors that were included or excluded from the analysis. One study included only CABG procedures whereas others excluded this procedure which is of significance as it is associated with the lowest incidence of AKI post-cardiac surgery^32^. The importance of comorbidities as confounders is further supported by superior predictive performance in paediatric cohorts, in which co-morbidities are almost invariably absent^17^.

The threshold level for pNGAL varied considerably across studies. Despite this limitation, results across similar studies were pooled since a standardised cut-off for pNGAL has not yet been defined. The variability in thresholds and diagnostic accuracy in the population may in part be due to the complex origin of NGAL. NGAL exists in at least three different molecular forms; a 25 kDa monomer, a 45 kDa homodimer, and a 135 kDa NGAL/matrix metalloproteinase-9 (MMP-9) covalently complexed heterodimer. The renal cells predominantly produce the monomeric form and to lesser extent the heterodimeric form, whereas neutrophils contain all molecular forms. The homodimer is, therefore, specific for neutrophils^33–35^. The systemic inflammatory response triggered by CPB will activate circulating neutrophils to release their granular contents, including NGAL. Indeed, NGAL concentrations have been shown to be correlated with CPB duration and furthermore it is the homodimeric form that predominates suggesting that neutrophils as opposed to renal cells are the main source^36^. Various commercially available NGAL assays were utilised in the studies included in this review, several of which were marketed as ‘research use only’ assays. The NGAL assays cannot distinguish between the molecular forms released by different tissues. There is also currently no standardisation of NGAL assays and the specificity of the assays for the monomeric form was not stated in manufacturer’s instructions for use.

The diagnostic utility of pNGAL is inherently flawed when assessed against an imperfect reference standard. Serum creatinine is diluted in fluid-loaded patients; therefore, the incidence of AKI may be underestimated in this cohort^37^. Elevated NGAL in the absence of creatinine-based criteria for AKI is associated with an increased requirement for RRT and mortality, but it is unclear whether this represents subclinical AKI or severity of the systemic inflammatory response^38,39^.

A large value of *I*^2^ was interpreted as meaning that the effect size varies substantively across studies. The *I*^2^ statistic merely designates the extent of inconsistency of findings across studies in the meta-analysis and reflects the extent to which confidence intervals from the different studies overlap with each other. Univariate tests for heterogeneity in sensitivity and specificity and the estimates of the *I*^*2*^ statistic are not recommended as they do not account for heterogeneity explained by phenomena such as positivity threshold effects^27^. A degree of heterogeneity is inevitable, and it could be argued that any degree of heterogeneity is acceptable provided the protocol is clearly defined and risk of bias has been assessed and findings interpreted considering this.

Tests for funnel plot asymmetry are designed primarily for use in randomized trials and should not be used in systematic reviews of diagnostic test accuracy as there is potential to incorrectly indicate publication bias^26^. A more appropriate method for detecting funnel plot asymmetry in reviews of diagnostic studies has been developed but also has low power when there is heterogeneity in the diagnostic odds ratio, as is present in this study^40^.

The hSROC curves appear to show optimisation of sensitivity at the expense of specificity at 4–8 h possibly in attempt to rule-in AKI earlier whereas at 24 h there is considerably more variability in sensitivity, perhaps indicating optimisation of specificity i.e., a rule out approach. It is noted that in the individual studies there is seldom an explanation of how the optimal cut-off point for pNGAL was reached. We suggest that it may be more appropriate to assess the utility of pNGAL as a rule out test in this population. The NICE diagnostics assessment programme manual however does not recommend the separate analysis of negative and positive predictive values as this approach fails to take into account the correlation between the two parameters. In addition, pooled analysis of NPV or PPV is not recommended because of the impact of disease prevalence on these parameters, which is likely to vary between studies^41^. Therefore, larger prospective studies or randomised controlled trials (RCTs) in a representative population are required.

### Limitations

The limitations of the meta-analysis are largely due to between study heterogeneity. Although risk of bias was assessed, and results of the meta-analysis were interpreted accordingly, the risk of bias in many cases was classified as unclear. Often measurement procedures performed within the studies differed significantly from those employed in routine practice, typically for reasons of pragmatism or cost. Many studies, for example, reported that samples were frozen and analysed as a single batch. The impact of analysing samples within a single batch is that it reduces variance increasing the likelihood of a significant finding. However, the results then may not translate to clinical practice, where samples are measured over many days and using different batches of reagents. The effect of freeze–thawing is also seldom stated, and this may be a potential source of a systematic increase or decrease in biomarker concentration. This could render clinical cut-off points invalid and lead to a higher FP or FN rate when introduced into routine practice. There was also, as previously discussed, significant heterogeneity in the characteristics of patients included in the studies.

Although the use of QUADAS-2 addresses the methodological issues concerning diagnostic accuracy, it does not address the issues associated with measurement. There are currently no guidelines available for evaluating the quality of measurement procedures in diagnostic accuracy studies. Therefore, this is an accepted limitation of the meta-analysis.

## Conclusions

Whilst there is a potential role for the diagnostic utility of pNGAL in this clinical setting, because of the limited number of studies, substantial heterogeneity between studies and large 95% confidence and prediction regions, reliable conclusions cannot be drawn. There is currently no standardisation of assays or thresholds, and the assays included in this analysis cannot distinguish between the various molecular forms of NGAL released by different tissues. Larger prospective studies or RCTs, ideally distinguishing the monomeric form of NGAL, in a population truly representative of those undergoing cardiac surgery requiring CPB, are required.

## Supplementary Information

Supplementary Information.

## Data Availability

All relevant data generated or analysed during this study are included in this published article.

## Abbreviations

AKI: Acute kidney injury
AKIN: Acute kidney injury network
AUC: Area under curve
CI: Confidence interval
CABG: Coronary artery bypass graft
CPB: Cardio-pulmonary bypass
DAP: Diagnostics Assessment Programme
FN: False negative
FP: False positive
hSROC: Hierarchical summary receiver operator characteristic
ICU: Intensive care unit
KDIGO: Kidney Disease: Improving Global Outcomes
LOS: Length of stay
NGAL: Neutrophil gelatinase-associated lipocalin
NICE: National Institute for Health and Care Excellence
NPV: Negative predictive value
PPV: Positive predictive value
QUADAS 2: Quality assessment of diagnostic accuracy studies version 2
ROC: Receiver operator characteristic
RIFLE: Risk, injury, failure, loss of kidney function and End-stage kidney disease
RRT: Renal replacement therapy
TN: True negative
TP: True positive
